# Self-Perception of Swallowing-Related Problems in Laryngopharyngeal Reflux Patients Diagnosed with 24-Hour Oropharyngeal pH Monitoring

**DOI:** 10.1155/2016/7659016

**Published:** 2016-02-04

**Authors:** Tamer A. Mesallam, Mohamed Farahat

**Affiliations:** ^1^Department of Otolaryngology, Head and Neck Surgery, King Saud University, Riyadh 11411, Saudi Arabia; ^2^Research Chair of Voice, Swallowing, and Communication Disorders, ORL Department, College of Medicine, King Saud University, Riyadh 11411, Saudi Arabia; ^3^Otolaryngology Department, College of Medicine, Al-Menoufia University, Shebin Al-Koum 32512, Egypt

## Abstract

*Background and Objectives.* Swallowing difficulty is considered one of the nonspecific symptoms that many patients with laryngopharyngeal reflux complain of. However, the relationship between laryngopharyngeal reflux and swallowing problems is not clear. The purpose of this work is to explore correlation between swallowing-related problems and laryngopharyngeal reflux (LPR) in a group of patients diagnosed with oropharyngeal pH monitoring and to study the effect of laryngopharyngeal reflux on the patients' self-perception of swallowing problems.* Methods.* 44 patients complaining of reflux-related problems were included in the study. Patients underwent 24-hour oropharyngeal pH monitoring and were divided into positive and negative LPR groups based on the pH monitoring results. All patient filled out the Dysphagia Handicap Index (DHI) and Reflux Symptom Index (RSI) questionnaires. Comparison was made between the positive and negative LPR groups regarding the results of the DHI and RSI ratings. Also, correlation between DHI scores, RSI scores, and pH monitoring results was studied.* Results.* Significant difference was reported between positive and negative LPR groups regarding DHI scores, RSI scores, and overall rating of swallowing difficulty. There was significant correlation demonstrated between DHI scores, RSI scores, and 24-hour oropharyngeal pH results.* Conclusion.* Laryngopharyngeal reflux appears to have a significant impact on patients' self-perception of swallowing problems as measured by DHI.

## 1. Introduction

Many laryngeal disorders have been attributed to laryngopharyngeal reflux (LPR) including reflux laryngitis, subglottic stenosis, laryngeal carcinoma, contact ulcers and granulomas, vocal nodules, and arytenoid fixation [1–4]. Symptoms of LPR include hoarseness, vocal fatigue, excessive throat clearing, globus pharyngeus, chronic cough, postnasal drip, and dysphagia [4]. In addition to voice problems, chronic cough, and throat clearing, swallowing difficulty was among the main complaints in patients with LPR and it has negative impact on their quality of life [5, 6].

Silbergleit et al. [7] developed the DHI, which is a patient-administered 25-item questionnaire that measures the handicapping effect of dysphagia on the emotional, functional, and physical aspects of the patient's life. DHI was translated into Arabic language and was found to be a valid and reliable tool for patients with oropharyngeal dysphagia including patients with LPR [8].

The oropharyngeal Dx-pH measurement system (Dx-pH; Restech Corp., San Diego, CA, USA) is considered a minimally invasive and relatively new device that recently has been used in many research studies concerning the diagnosis of LPR. The device has been reported to be reliable in the detection of acidic reflux events in the posterior oropharynx [9–11].

The aim of this study was to explore correlation between swallowing-related problems and laryngopharyngeal reflux (LPR) in a group of patients diagnosed with oropharyngeal pH monitoring and to study the effect of laryngopharyngeal reflux on the patients' self-perception of swallowing problems.

## 2. Patients and Methods

### 2.1. Subjects

The Institutional Review Board at College of Medicine has approved the study proposal. The study included 44 patients who were referred to the Reflux Clinic to perform 24-hour oropharyngeal pH monitoring test. Adult male and female patients complaining of reflux-related symptoms were included in the study. LPR-related complain included change of voice character, frequent throat clearing, foreign body sensation, or cough. Patients with a reported swallowing difficulty related to neurological or structural problems were excluded from the study.

### 2.2. DHI and RSI Rating

All patients in the study were instructed to fill out the DHI and RSI before being admitted to the clinic. Patients were asked to rate the questionnaires precisely according to their current condition. Patients rated their swallowing problems in the DHI according to the suggested rating of “never, sometimes, or always.” Ratings were scored considering 0 for never, 2 for sometimes, and 4 for always. Also, patients were asked to rate the overall severity of their swallowing difficulty on a scale from 1 to 7 given that 1 refers to normal or no problem while 7 refers to a severe problem. Similarly, patients were instructed to fill out the 9-item RSI on a rating scale ranging from 0 to 5, where 0 means no problems and 5 means a severe problem. Patients were given clear instructions about how they will fill out the 2 questionnaires according to the above-mentioned rating scales.

### 2.3. 24-Hour Oropharyngeal pH Monitoring

The diagnosis of laryngopharyngeal reflux in the study group was confirmed using 24-hour oropharyngeal Dx-pH probe system (Restech Corp., San Diego, CA). The patients were instructed to keep their normal daily activity as usual during the study and were given a diary to record the meal times and recumbent position times.

Following the 24-hour recording, analysis of the data was carried out using a software system provided with the device. Acidic reflux thresholds were set for 5.5 in the upright position and 5.0 in the supine position; meanwhile the meal times have been excluded from the analysis. The system automatically generates the Ryan score, which is a composite score, calculated based on the given pH thresholds of upright and supine positions. The score incorporates three main parameters including number of reflux episodes, the duration of longest reflux episode, and the percentage of time below the predetermined pH threshold. Scores greater than 9.41 in the upright position and/or 6.80 in the supine position were considered suggestive of LPR [9, 12, 13].

### 2.4. Statistical Analysis

Patients were divided into positive and negative LPR groups according to the results of the oropharyngeal pH monitoring. Comparisons were made between the positive and negative LPR groups regarding the results of the DHI, RSI, and overall rating of swallowing difficulty. Correlation was studied between the results of oropharyngeal pH-related measures and DHI results.

As a prerequisite of our statistical analysis, a numerical assessment of the normality of data was undertaken. Kolmogorov test was done to test the normal distribution of the data. Based on the normality test, all data were not normally distributed. Thus, nonparametric statistical analyses were applied. Spearman's correlation coefficient was used to test the correlation between variables while Mann-Whitney test was used for comparison of the DHI, RSI, and overall swallowing difficulty rating results among the study groups. The level of significance was set as *P* value <0.05. The Statistical Package for the Social Sciences, Version 22 (SPSS Inc, Chicago, IL, USA) was used for all statistical analysis.

## 3. Results

The study included 44 subjects in the age range between 22 and 67 years old with a mean age of 45.65 years old. There were 19 male and 25 female subjects who participated in the study. Based on the 24-hour oropharyngeal pH monitoring results, 26 patients were diagnosed with positive LPR diagnosis while 18 patients had negative LPR diagnosis.  Table 1 shows age and gender distribution of the study subjects among positive and negative LPR groups.

On comparing the DHI scores between positive and negative LPR groups, positive LPR group reported significantly higher DHI scores than that of the negative LPR group (*P* < 0.01). Similarly, there were significantly higher ratings for the overall swallowing difficulty in the positive LPR group compared to the negative one (*P* < 0.001). Moreover, positive LPR group showed significantly higher RSI scores compared to the negative LPR group (Table 2).

As shown in Table 3, significant positive correlation was reported between the DHI total score and RSI score, number of reflux episodes, and the Ryan score (*r* ranged from 0.3 to 0.68). Also, there was significant positive correlation between the overall swallowing difficulty and RSI score, number of reflux episodes, and Ryan score (*r* ranged from 0.41 to 0.52).

## 4. Discussion

Dysphagia has been frequently mentioned in the literatures as one of the presentations of LPR. Moreover it has been reported that it negatively affects the patients' quality of life [2, 14–19]. The aim of this work was to study the effect of LPR on patients' self-perception of swallowing difficulties as measured by DHI. Also, to explore the correlation between LPR and swallowing-related problems in a group of LPR patients diagnosed with oropharyngeal pH monitoring.

The DHI has been developed to measure patients' rating of their swallowing problems. In this study DHI scores were significantly higher in LPR positive group compared to the negative LPR group. Also, the overall rating of swallowing difficulty was significantly higher in positive LPR group compared to the negative one. These findings indicate that LPR has a negative effect on the patients' self-perceived swallowing difficulty. These results confirm the findings of Aviv et al. [20], as they have suggested that anatomical and physiological changes in the hypopharynx related to LPR contribute to significant abnormalities in swallowing including penetration and aspiration. They also reported that treatment of LPR with proton pump inhibitors significantly reduced the number of aspiration and penetration events.

In this study, we assessed LPR from both subjective and objective point of view. Objectively, we used the oropharyngeal pH monitoring to classify patients into positive and negative LPR groups while RSI results represent the subjective impression of patients regarding their LPR problem. Not only DHI results were higher in the positive LPR group but also RSI rating was significantly higher compared to the negative group. This suggests that LPR does not only negatively affect the DHI rating but also has similar effect on RSI results. These results emphasize the significant influence of LPR on patients' quality of life and coincide with the findings of other related studies assessing the quality of life of patients with LPR [5, 6].

The positive correlation that has been reported between the DHI scores and both RSI and pH monitoring results (including number of reflux episodes and Ryan score) emphasizes the negative influence of LPR on patients' self-perception of swallowing problems. Interestingly, the rating of overall feeling of swallowing difficulty also showed significant positive correlation with the RSI scores and pH monitoring results. This signifies that apart from sophisticated dysphagia assessment tools, general feeling of swallowing difficulty is also negatively affected by LPR. These collective results reflect the negative effect of LPR on the overall swallowing quality of life issues of patients with such a problem.

## 5. Conclusion

The results of this study indicate that overall feeling of swallowing difficulties is significantly higher in LPR patients. There appears a negative effect of LPR on patients' self-perception of swallowing-related problems as measured by DHI.

## Figures and Tables

**Table 1:** Age and gender distribution among the positive and negative LPR groups.

	LPR positive group (*N* = 26)	LPR negative group (*N* = 18)
Patients (*n*)	26	18
Age (mean ± SD)	44.80 ± 12.99	46.88 ± 10.19
Gender		
Male *n*, (%)	10 (26%)	9 (50%)
Female *n*, (%)	16 (74%)	9 (50%)

**Table 2:** Comparison between LPR positive and negative groups regarding the DHI and RSI scores.

	LPR positive group (*N* = 26)		LPR negative group (*N* = 18)		Mann-Whitney *P*
	Mean	SD	Mean	SD	
DHI	33.46	17.88	19.44	22.26	<0.01
Overall swallowing difficulty	3.76	1.14	2.11	1.02	<0.0001
RSI	22.69	5.88	11.55	10.43	<0.0001

**Table 3:** Correlation between the DHI, RSI, and the pH monitoring results.

	RSI	Number of reflux episodes	Ryan score
Spearman's rho			
DHI			
Correlation coefficient	0.68^*∗∗*^	0.33^*∗*^	0.30^*∗*^
Sig. (2-tailed)	0.001	0.02	0.04
*N*	44	44	44
Overall swallowing difficulty			
Correlation coefficient	0.52^*∗∗*^	0.45^*∗∗*^	0.41^*∗∗*^
Sig. (2-tailed)	0.0001	0.002	0.005
*N*	44	44	44

^*∗∗*^Correlation is significant at the 0.01 level (2-tailed).

^*∗*^Correlation is significant at the 0.05 level (2-tailed).
